# Phenotypic Variants of Staphylococci and Their Underlying Population Distributions Following Exposure to Stress

**DOI:** 10.1371/journal.pone.0077614

**Published:** 2013-10-18

**Authors:** Laura A. Onyango, R. Hugh Dunstan, Timothy K. Roberts, Margaret M. Macdonald, Johan Gottfries

**Affiliations:** 1 Environmental and Pathogenic Microbiology Laboratory, School of Environmental and Life Sciences, University of Newcastle, Newcastle, New South Wales, Australia; 2 Department of Chemistry, Gothenburg University, Göteborg, Sweden; Rockefeller University, United States of America

## Abstract

This study investigated whether alterations in environmental conditions would induce the formation of small colony variant phenotypes (SCV) with associated changes in cell morphology and ultra-structure in *S. aureus*, *s.* epidermidis, and *S. lugdunensis*. Wild-type clinical isolates were exposed to low temperature (4°C), antibiotic stress (penicillin G and vancomycin; 0-10,000 µg mL^-1^), pH stress (pH 3-9) and osmotic challenge (NaCl concentrations of 0-20%). Changes in cell diameter, cell-wall thickness, and population distribution changes (n ≥ 300) were assessed via scanning and transmission electron microscopy (SEM and TEM), and compared to control populations. Our analyses found that prolonged exposure to all treatments resulted in the subsequent formation of SCV phenotypes. Observed SCVs manifested as minute colonies with reduced haemolysis and pigmentation (NaCl, pH and 4°C treatments), or complete lack thereof (antibiotic treatments). SEM comparison analyses revealed significantly smaller cell sizes for SCV populations except in *S. aureus* and *S. epidermidis* 10% NaCl, and *S. epidermidis* 4°C (p<0.05). Shifts in population distribution patterns were also observed with distinct sub-populations of smaller cells appearing for *S. epidermidis*, and *S. lugdunensis*. TEM analyses revealed significantly thicker cell-walls in all treatments and species except *S. lugdunensis* exposed to 4°C. These findings suggest that staphylococci adapted to environmental stresses by altering their cell size and wall thickness which could represent the formation of altered phenotypes which facilitate survival under harsh conditions. The phenotypic response was governed by the type of prevailing environmental stress regime leading to appropriate alterations in ultra-structure and size, suggesting downstream changes in gene expression, the proteome, and metabolome.

## Introduction

Variability within microbial populations is not a new concept in the field of microbiology. It has been described from as early as the 19^th^ century although its role within such populations was not clearly understood at the time [1]. With increasing reports of deviations within classical bacterial forms, research into the function of microbial variants revealed variations in morphological, immunological, and physiological parameters [2,3] that may contribute to their persistence, virulence and consequent survivability. One of the more interesting variants described over the past decade has been the small colony variants (SCV), a bacterial sub-population that exhibits atypical growth features compared to their parental wild-type (WT) population. More importantly, collective reports and research have linked bacterial SCVs to several recurring infections that are intractable to conventional treatment regimes [4].

Staphylococci are one of the more commonly involved nosocomial species associated with recurring disease states. WT staphylococci have been shown to be capable of growing over a wide range of environmental conditions inferring a capability to rapidly adapt their physiology for survival and take opportunistic advantage when optimal conditions prevail [5]. SCV have frequently been isolated from both clinical and laboratory settings under sub-optimal conditions [6–9]. Although these variants were known to be a naturally occurring part of the bacterial life cycle, interest in their physiology and function only escalated when they were associated with persistent clinical infections [10–12]. Several morphological differences have been described for these variants including variation in colony size, pigmentation and haemolytic irregularities [13–16]. Investigations have revealed some understanding of the mechanisms used to sustain persistent infections [17–19], some of which are related to these morphological variations.

The *in vivo* formation of staphylococcal SCVs is widely documented and several factors including antibiotic administration combined with intracellular factors have been credited for the selection of this phenotype [20–22]. The majority of the *in vitro* investigations into SCV formation have involved exposure of *S. aureus* isolates to antibiotic therapy [9,23,24]. The first aim of this study was therefore to determine whether the formation of SCVs was a general response mechanism for survival or whether it was a specific adaption to counter exposure to antibiotic challenge. To investigate this hypothesis, a range of environmental stress conditions were used including antibiotics penicillin G (Pen G) and vancomycin (VA), acidic and alkaline pH, low temperature, and osmotic stress. The capacities to form SCVs in the coagulase-negative staphylococcal (CNS) species *S. epidermidis* and *S. lugdunensis* were also investigated. Since SCV colonies have slower growth rates and represent a relatively smaller biomass in comparison to the WT colonies, it was proposed that SCV cells would be smaller and possess structural alterations when compared with their corresponding control cells. The second aim was therefore to determine whether morphological differences existed in populations of SCV cells compared with populations of their corresponding control cells. Cell populations within the SCV and control colonies were assessed for ultra-structural differences including changes in cell size distributions and cell-wall thickness characteristics by use of scanning and transmission electron microscopy (SEM andTEM).

## Materials and Methods

### Bacterial growth

*S. aureus*, *S.* epidermidis, and *S. lugdunensis* were isolates derived from an earlier investigation [25] and maintained as culture stock within the laboratory. Briefly, clinical swabs were obtained from various anatomical sites and cultured onto Columbia horse blood agar (HBA), then identified for morphological and biochemical characteristics. The isolates were appropriately stored and routinely sub-cultured onto HBA to maintain viability. Identity checks were performed regularly by PCR. Cultures of 100mL were grown in 250mL conical flasks to mid-exponential phase and then harvested. Unless otherwise mentioned, harvesting was done by centrifugation and the resulting cell-pellets washed and re-suspended in sterile water as appropriate.

### Treatments

A series of treatments were applied to cultures of *S. aureus*, *S.* epidermidis, and *S. lugdunensis* including exposures to either 4°C, antibiotics (VA and Pen G), pH or osmotic stresses with replication at n=9 to ensure appropriate reproducibility. To assess for growth under low-temperature challenge, a revised methodology [16] was used for the purposes of this study. In summary, replicate cultures of each species were grown in BHI media to mid-exponential phase at 37°C and thereafter incubated at 4°C for 8weeks. Samples of the cold exposed cultures were aseptically removed and diluted ten-fold before plating (5 µL) in triplicate on HBA plates. Resulting SCV colonies were further inoculated onto a second generation of HBA plates, incubated at 37°C for 24hrs and assessed for pigmentation and haemolysis. For antibiotic challenge, a double isolation method was used as described by Seaman et al [26] with some modification. Replicate cultures of all three species were grown to mid-exponential phase in Mueller-Hinton broth (MHB) at 37°C and then harvested without washing. The resulting cell-pellet was aseptically re-inoculated into fresh pre-warmed MHB adjusted to 0.01, 0.1, 1.0, 10, 100, 1000, and 10,000 µg mL^-1^ of either Pen G or VA and incubated for 5hrs at 37°C. Cells were harvested and 100 µl plated onto MH agar plates supplemented with corresponding antibiotic concentrations for up to 72hrs at 37°C. Individual SCV colonies were sub-cultured onto HBA plates for assessment of pigmentation and haemolysis. For osmotic stress, a starter culture was grown in nutrient broth (NB) to mid-exponential phase at 37°C. Cells were harvested without washing and inoculated into fresh NB supplemented with NaCl (0, 5, 10, 15 and 20%). Cultures were grown for 5hrs at 37°C, harvested and 100 µl aliquots sub-cultured onto the corresponding NaCl agar concentrations and incubated at 37°C. Resulting SCV colonies were sub-cultured onto HBA plates for assessment of pigmentation and haemolysis. The effect of pH stress was evaluated by growing cultures in tryptone soya broth (TSB) to mid-exponential phase at 37°C. Cells were harvested without washing and the resulting cell-pellet inoculated into fresh TSB adjusted to pH values of 3, 5, 7, and 9. Cultures were grown for 5hrs, harvested and 100 µl aliquots plated onto tryptic soya agar plates adjusted to the corresponding pH levels. Resulting SCV colonies were sub-cultured onto HBA plates for assessment of pigmentation and haemolysis. Control cultures were grown concurrently at 37°C with each of the treatments.

### SCV characterization

Respective plate cultures were assessed 24 hrs post-incubation and colonies categorized based on size, haemolysis (on HBA) and pigmentation. Those that were >1mm in diameter, pigmented, and haemolytic were recorded as WT population. SCVs were recorded as being <1mm in size, with reduced pigmentation and haemolytic activity as described in literature [13,15,19]. Cultures exposed to antibiotic stress were assessed 48-72hrs post-incubation.

### Reversion

SCVs generated from treatment plates were tested for reversion by sub-culturing individual colonies (n =9) onto HBA plates overnight under optimal conditions (37°C, no stress). Reversion was defined as the capacity of the SCV colonies to revert to WT cultures following subsequent plating and growth under optimal conditions. Colonies that generated WT characteristics (>1mm, pigmented, and haemolytic in comparison to control plates) were recorded as exhibiting reversion.

### Species identification

Both WT and SCV colonies were analysed by API® Staph test (bioMérieux) and PCR of the 16S rRNA gene method [27] to ascertain the purity of cultures and confirm the identity of isolates.

### Determination of MIC

This was done by the broth micro-dilution technique [28] guidelines. The MIC value was recorded as the lowest antibiotic concentration at which there was no visible bacterial growth.

### Sample preparation for SEM and TEM analyses

Colonies (control and resulting SCV colonies) obtained post-incubation were prepared for SEM and TEM analyses. For SEM analysis, smears were prepared by gently suspending each colony type in a drop of sterile water on a 13 mm glass cover-slip. These were air-dried, washed for 5 sec with ethanol/acetone solution, and then rinsed using sterile water. Samples were allowed to air dry and stored in a desiccator overnight before sputter coating with 10 nm gold and viewed under a scanning electron microscope (Philips XL-30 Model Phillips + Oxford ISIS EDS) at 15 kV. Cell-diameter measurements were performed on cellular populations derived from both control and SCV colonies. In these analyses n=300 cells were examined in order to ascertain that the differences observed were not artefacts of the preparation process. Samples for TEM analyses were prepared according to the method described in [16]. Cell-wall thickness measurements were performed for cellular populations of both the control and SCV colonies and at least five to ten different fields of view were examined and n=300 cells assessed to ascertain that the characteristics observed were not artefacts of the preparation process.

**Figure 1:**
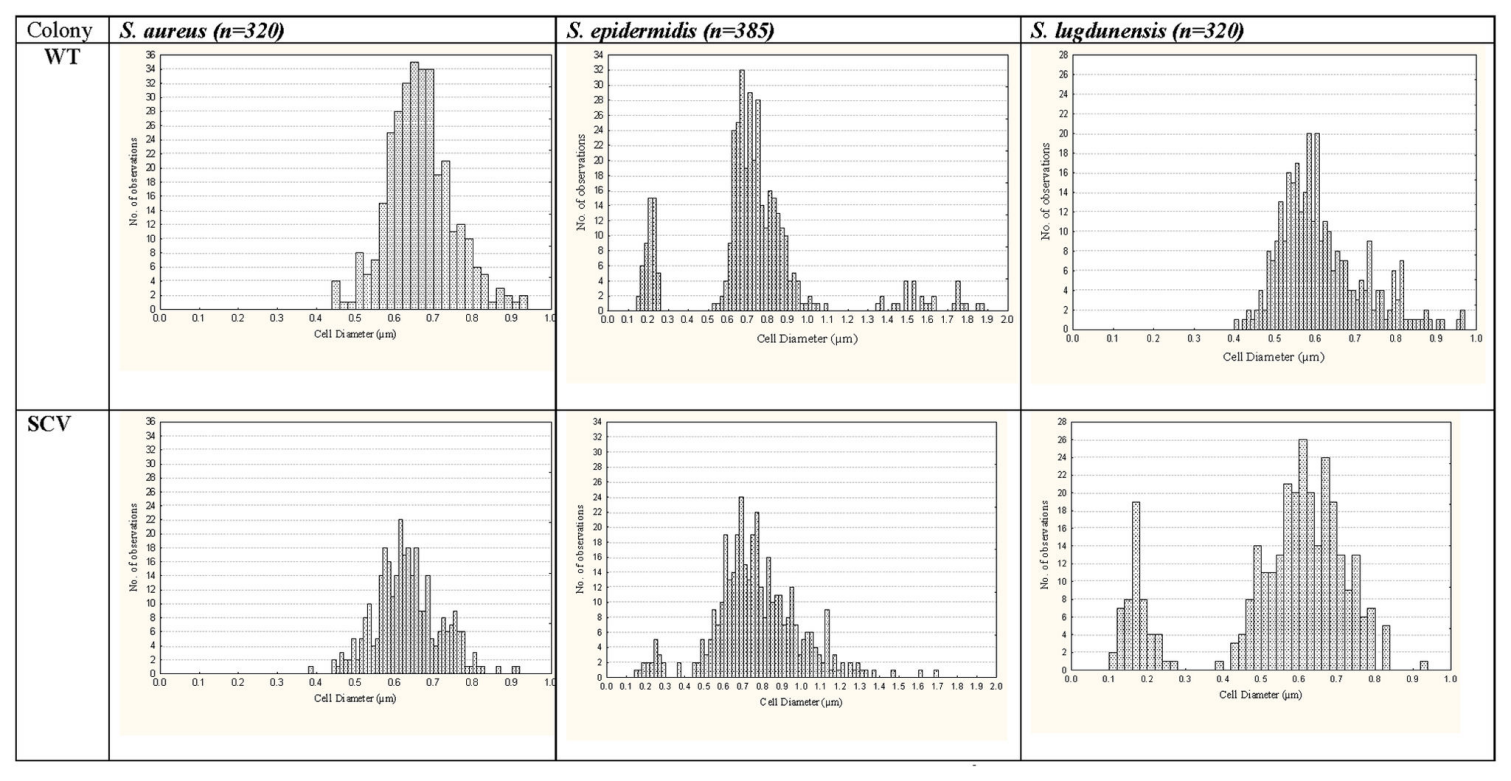
Population distributions of cell sizes from WT colony and corresponding SCV colony cells (4°C) of *S. aureus*, *S.* epidermidis and *S. lugdunensis* which have been ranked on the basis of their cell diameter measurements following SEM analyses.

### Statistical analysis

All experiments were performed in triplicate (n=9) to ensure reproducibility, and data presented as the mean ± SD of replicate results. UTHSCSA Image Tool™, (Version 3) was used to measure cell diameters and cell-wall thickness. Data was analysed by ANOVA using Statistica TM (Version 6.1, Statsoft, Tulsa, OK).

**Figure 2:**
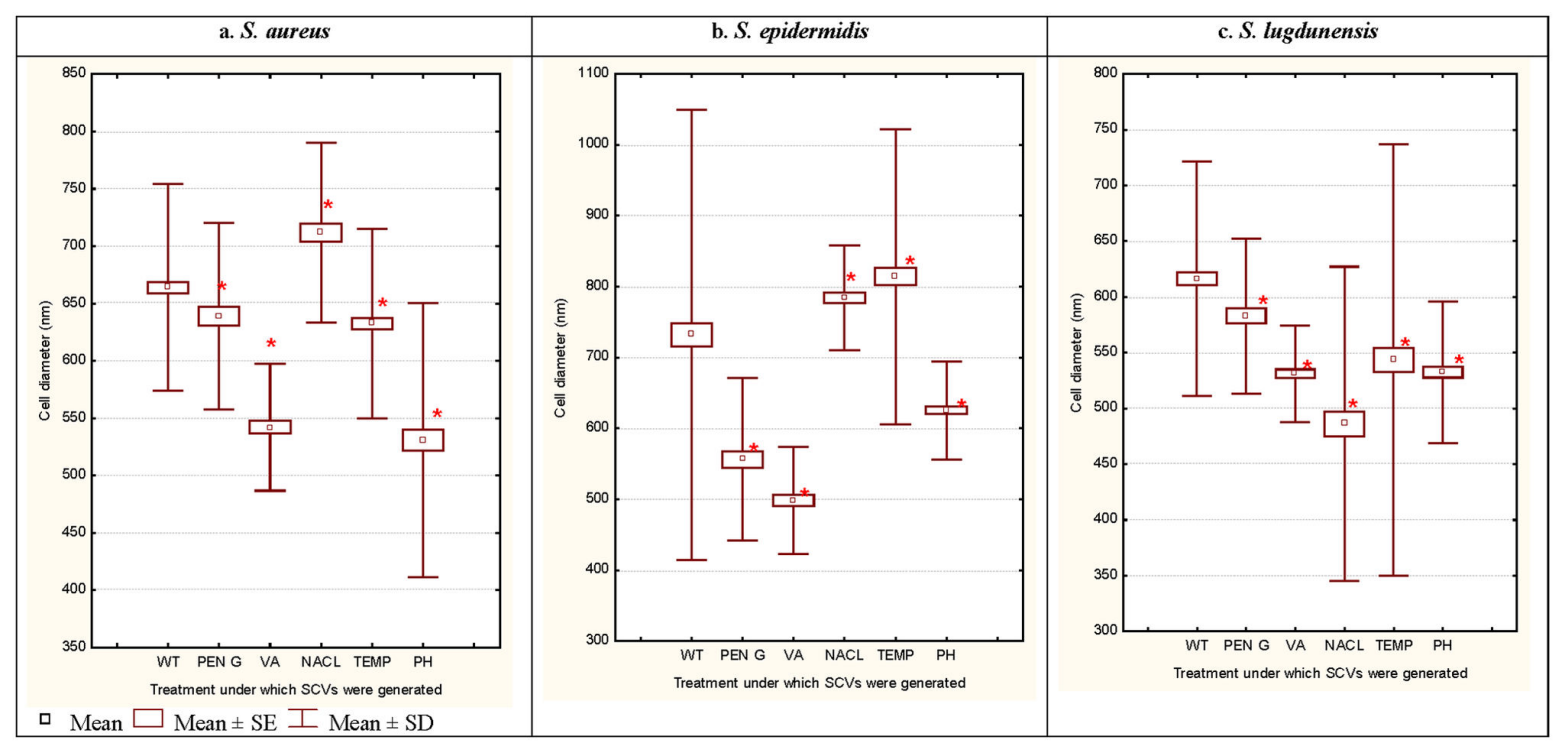
Mean cell sizes assessed by SEM (n>300) of WT cells and corresponding SCV cells of *S. aureus* (a), *S. epidermidis* (b) and *S. lugdunensis* (c). SCV cells were generated following exposure to the antibiotics penicillin G (Pen G) and vancomycin (VA) (random antibiotic concentrations generating SCV were used), 10% NaCl, 4°C temperature and pH 5 stresses. Asterisk (*) indicates significant differences compared with corresponding WT cells (P<0.05).

## Results

### SCV formation in response to environmental stresses

Cultures of *S. aureus*, *S.* epidermidis, and *S. lugdunensis* were grown in BHI broth cultures to the mid-exponential phase and then subjected to prolonged exposure to 4°C for 8 weeks. When diluted samples of these cold-treated cultures were spread on HBA plates and subsequently grown at 37°C for 24 hours, significant increases in the incidence of SCV colonies were observed compared with the respective control cultures. The incidences of SCV in treatment sub-cultures were *S. aureus* at 39% whereas *S. epidermidis* and *S. lugdunensis* both had SCV’s at >75% of the total colonies. The SCV colonies observed were <1mm in size, with the majority showing reduced pigmentation and haemolytic activity in comparison to their corresponding control cells. A small number of non-pigmented, non-haemolytic SCVs were also observed but with low reproducibility. SCV colonies tested for reversion following prolonged exposure to 4°C were capable of generating WT colonies on HBA plates (24 h at 37°C) and speciation was confirmed by PCR.

Cultures of *S. aureus*, *S.* epidermidis, and *S. lugdunensis* exposed to 10% and 15% added NaCl yielded SCV’s following subculture onto HBA plates. These small colonies were slightly pigmented with reduced haemolytic activity. All tested SCV colonies were capable of generating WT colonies following sub-culture onto HBA plates for 24 h at 37°C and their identities were confirmed by PCR. Cultures of *S. aureus*, *S.* epidermidis and *S. lugdunensis* exposed to pH 5 stress yielded SCV growth following sub-culture onto HBA plates. SCV colonies arising from this treatment also exhibited both reduced haemolysis (on HBA) and pigmentation. SCV colonies tested for reversion were capable of generating WT colonies following sub-culture onto HBA plates and their identities were confirmed by PCR. Exposure to pH 3 did not support any obvious growth of cells and exposures to pH 7 and 9 did not result in any SCV formation.

The three staphylococcal species were evaluated for their antibiotic resistance characteristics and the following MIC values (µg mL^-1^) were recorded: *S. aureus* - VA 7.8; Pen G 15.6; *S. epidermidis* - VA 3.9; Pen G 1300; and *S. lugdunensis* - VA 10.4; Pen G 11.7. Cultures of staphylococci exposed to the lower antibiotic concentrations (0.01 and 0.1 mg mL^-1^) of either Pen G or VA, had prolific WT growth and no SCVs were observed. SCVs were observed for *S. aureus*, *S.* epidermidis and *S. lugdunensis* at VA exposures of 1, 10 and 100 mg mL^-1^. For Pen G treatments, SCVs were observed for *S. aureus* and *S. lugdunensis* after exposure to 1, 10 and 100 mg mL^-1^. *S. epidermidis* displayed a very high resistance to the Pen G (MIC=1300 µg mL^-1^) with SCVs not appearing until exposure levels of 1,000 and 10,000 mg mL^-1^. Most of the antibiotic-induced SCVs were primarily non-pigmented and non-haemolytic (on HBA), although minute (<1mm) slightly pigmented and haemolytic colonies were also observed in some replicate plates, albeit in lower numbers. All sampled small colonies formed following exposures to VA and Pen G were shown to generate WT colonies after at least two passages in stress-free media (HBA, 37°C). There were no distinct morphological differences between SCV generated from the different antibiotic concentrations. All concentrations of antibiotics tested showed the same results and random samples were used for the proceeding EM analyses.

### Ultra-structure analyses

Smear samples of individual SCV colonies derived following exposures to critical stress regimes (VA, Pen G, 4°C, 10%NaCl and pH5) and their corresponding control colonies were prepared for examination by SEM. Multiple micrographs were taken for each of the 9 replicates for all control and SCV treatments to enable random measures of cell diameters for cells (n>300) in each treatment category. The data for these evaluations have been presented in Figure 1 as detailed population distributions for the WT from control cultures and the corresponding SCVs of *S. aureus*, *S.* epidermidis and *S. lugdunensis* formed in response to prolonged exposure to 4°C. The distributions for WT populations show substantial diversity within all the staphylococcal populations, with *S. epidermidis* appearing to have at least 3 clusters of cell types based on the cell diameter measures. The cell size distribution patterns altered in the corresponding SCV populations following exposure to 4°C treatment, with *S. lugdunensis* populations appearing to form a subgroup of smaller sized cells which was not evident in the WT distribution. This change in population dynamics would be missed if the data were only presented as mean +/- SD.

**Figure 3:**
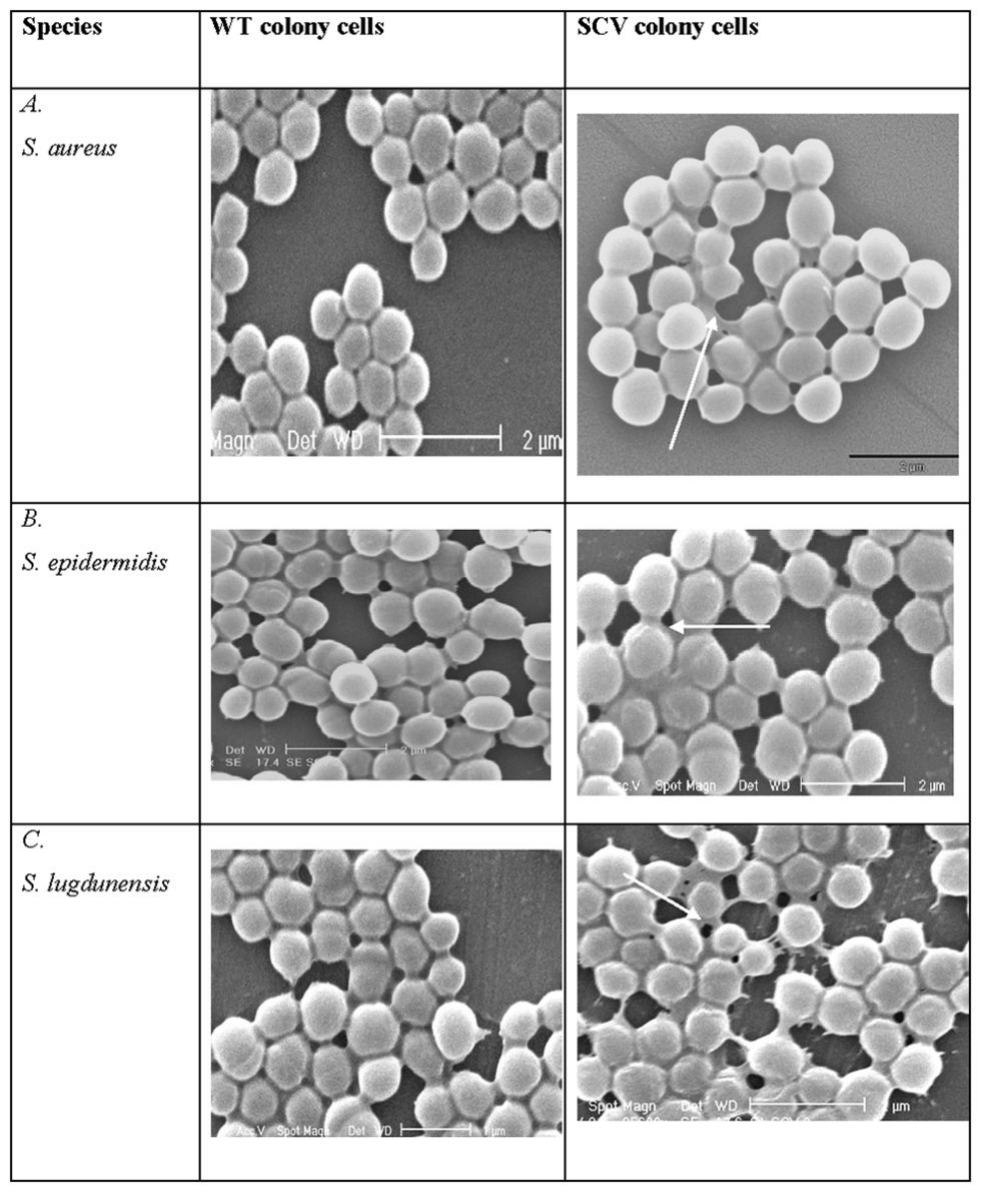
SEM images of *S. aureus*, *S.* epidermidis and *S. lugdunensis* WT and their vancomycin (VA) -induced (100 µg mL^-1^) SCVs: SEM images with SCV cells displaying a more prevalent extracellular matrix material (arrow) than their corresponding WT cells.

Shifts in cell-size distribution patterns were observed for all treatments across all three species (data not shown but have been provided as Table S1; Figures S1-S3 in File S1). The 10% NaCl treatments of *S. aureus* and *S. epidermidis*, as well as 4°C treatment of *S. epidermidis*, resulted in SCV cells with significantly larger mean cell sizes compared with their corresponding control cells. All other treatments resulted in significantly smaller cell sizes compared with their corresponding control cells for *S. aureus*, *S.* epidermidis and *S. lugdunensis* as shown in Figure 2. Additional SEM analyses of *S. aureus*, *S.* epidermidis and *S. lugdunensis* revealed the presence of an extracellular matrix material in both control and SCV cell populations (Figure 3a; *S. lugdunensis* samples shown). Extracellular matrix material appeared to be more prevalent amongst the SCV cells compared with their corresponding control cell preparations (indicated by arrow) but it was not possible to quantify this material for comparison.

**Figure 4:**
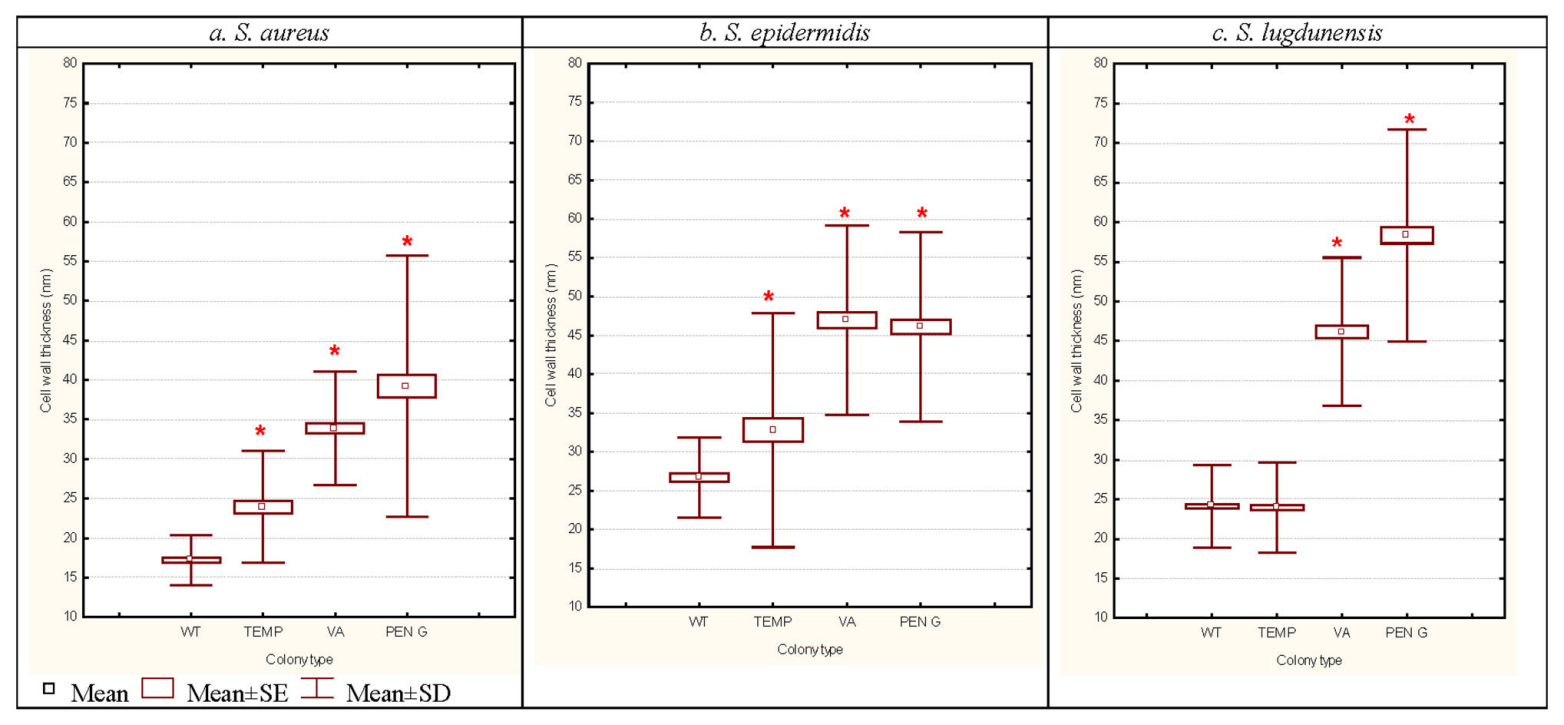
Comparisons of mean cell-wall thickness (nanometres) from SCV cells generated following exposures to 4°C and antibiotics (VA and Pen G; random concentrations utilised) in comparison with their corresponding WT cells taken from *S. aureus*, *S.* epidermidis, and *S. lugdunensis* samples and examined under TEM (n=300). Asterisk (*) indicates significant differences compared with corresponding WT cells (P<0.05).

TEM analyses revealed unique and consistent ultra-structural changes in SCV cells associated with cell-wall composition and thickness (Figure 3b). TEM images of *S. epidermidis* have been used as a representation of these characteristics where it is visually evident that the cell-wall is more diffuse and thicker in the SCV cells compared with corresponding cells from the control cultures. Cell-wall thickness measures were recorded for the comparisons between control and antibiotic and temperature-treated cells of all three staphylococci (n=300) and the comparisons of these data have been summarised in Figure 4. It was interesting to note that the WT coagulase negative staphylococci *S. epidermidis* and *S. lugdunensis* had significantly thicker cell-walls than the coagulase positive *S. aureus* (p<0.05). Prolonged exposure to 4°C resulted in significantly thicker cell-walls in the SCV cells compared with the corresponding control for *S. aureus* and *S. epidermidis* but not *S. lugdunensis*. Exposure to VA and Pen G resulted in an approximate doubling of cell-wall thickness for all three staphylococci.

## Discussion

This investigation revealed that *S. aureus*, *S.* epidermidis and *S. lugdunensis* were capable of yielding SCV phenotypes following exposures to pH5, 10%NaCl, 4°C and the antibiotics vancomycin and penicillin G. The finding that SCVs of *S. aureus* and the two coagulase negative staphylococci (CNS) appeared following these different treatments suggested that this phenotypic switch could be a common stress response to provide a more resilient form of staphylococci with altered metabolic rates and profiles. Differences noted between SCV populations formed after antibiotic exposure in comparison to the other stressors supported alterations in metabolism. Non-haemolytic and non-pigmented colonies were common characteristics reported by several other SCV researchers where antibiotics have been used [17,19,29] and it was suggested that these responses to antibiotics affected pathways involved in pigment and haemolysin production. While haemolysis and pigmentation may not be substantial *in vitro*, a down-regulation of these factors *in vivo* may account for the less virulent and chronic nature associated with SCV-related infections [17,20]. The different phenotypic profiles suggested that perhaps unique SCV phenotypes exist determined by the nature of the stresses applied.

**Figure 5:**
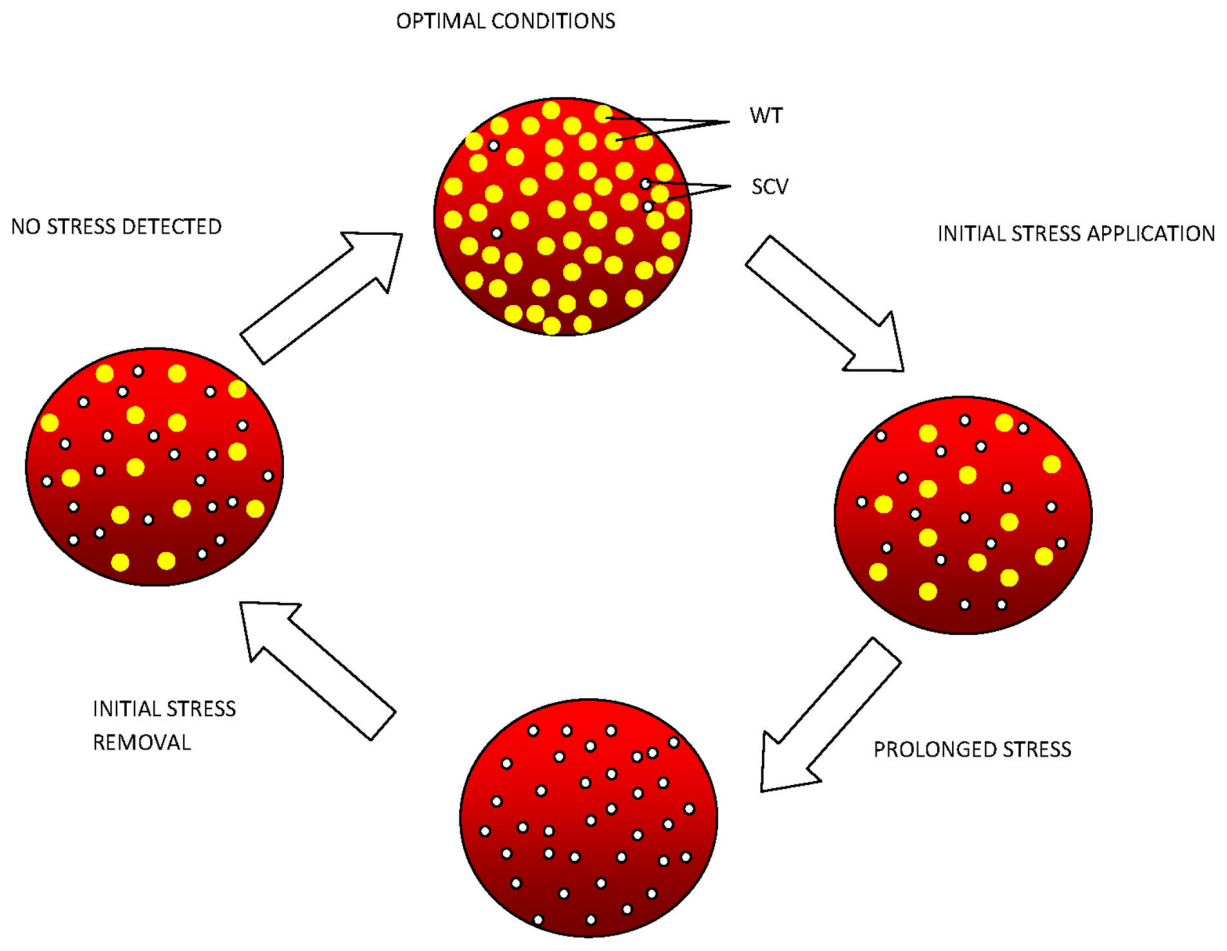
Diagram scheme indicating possible stages of wild-type-SCV life/stress cycle. Under optimal conditions, WT populations prevail, perhaps masking the SCV phenotype. Introduction of stress selects for a more resilient phenotype, changing the population dynamics from WT prevalence to SCV prevalence which persist even under prolonged exposure to stress. Removal of stress shifts population dynamics with WT populations dominating again.

Following the emergence of SCV colonies in response to the various stresses, colonies were sub-cultured under stress-free conditions to assess the stability of this phenotype. The re-appearance of WT colonies from sub-cultured SCV colonies was indicative of a reversion capability. *S. aureus* SCVs have been shown previously to cycle between WT (absence of stress) and SCV (presence of stress) phenotypes [9]. Similar observations in this study involving both *S. aureus* and the CNS species supported the hypothesis that the formation of SCVs represented a transient phenotypic response to stress, possibly involving epigenetic control to facilitate rapid survival in the presence of adverse conditions. Moreover, the ability of the sub-cultured SCV colony to generate both WT colonies and some SCV colonies in subsequent stress-free HBA plate cultures further supported the concept of epigenetic involvement and the notion that bacterial cultures exist as heterogeneous populations. The dominating phenotypes in the culture populations would therefore be dependent on the prevailing environmental conditions as summarized in Figure 5.

SEM analyses revealed significant alterations in cell sizes between control and SCV cellular populations. The exposures of *S. aureus* and *S. epidermidis* to 10%NaCl, and *S. epidermidis* to 4°C resulted in an increase in bacterial cell diameters compared with their controls (Figure 2). This was consistent with prior reports observing increased mean cell sizes when *S. aureus* cells were grown in the presence of osmotic stress where they grew slower than control samples but also showed retarded cell separation [30,31]. Slow growth in stressed cultures would consequently affect the rate of cell division that is triggered when a cell reaches critical mass for division, resulting in a decrease in the density of the cells within a colony and would account for the substantially smaller colony sizes observed in SCVs compared to their corresponding control colonies.

All other treatments resulted in significant reductions in cell size as shown in Figure 2. These data suggested that the SCV response is not the same for all environmental stress conditions. The colony morphology of these adaptive responses may yield similar colony morphologies but the cells would have different structural and physiological adaptations in place. It was also evident that the different staphylococcal species had different adaptive mechanisms for the same environmental stresses. For example, SCVs from *S. aureus* and *S. epidermidis* displayed significantly larger cell diameters in response to 10%NaCl exposure, but *S. lugdunensis* displayed a significantly reduced cell diameter following the same treatment. One explanation for decreased cell sizes may be provided by a reduction in metabolic rate leading to smaller cells. Studies have shown that bacterial cell size changes with varying environmental conditions and, under most of these stresses, the cell sizes were significantly smaller [32]. In such instances, the small cell sizes have been related to slow growth and proposed to be advantageous to these bacterial populations in the face of challenging conditions [32–34].

In clinical settings, slow growth *in vivo* is important as a survival technique and the capacity of staphylococci to switch to an SCV type of phenotype with slow growth may represent an important attribute for sustainability within a host. Once conditions are conducive again, they can switch to the more virulent and aggressive phenotype with fast growth capacity to invade more tissues. This cycling between phenotypes would result in a poor clinical outcome but represent excellent survival features for the bacterium.

Analyses of population distributions between control and SCV cells provided further insight into the capacity for survival of staphylococci by demonstrating diversity within their populations. A closer examination of the population distribution graphs (Figure 1) showed that the populations were not always normally distributed in the control colonies. The distribution patterns could be skewed or contain subgroups of cell sizes. The key finding in this study was that the distribution patterns for the SCV populations altered substantially for the CNS compared with their control populations. It was evident that *S. epidermidis* and *S. lugdunensis* SCVs had distinct sub-populations of significantly smaller cells. Supporting the observation of smaller subgroups of cells, viable cells were observed in sterile filtrates of all three staphylococci held under various stress conditions following filtration of cell preparations through 0.45µm and 0.22µm sterile filters [35]. The goal of population diversity would be to ensure that various phenotypes were present at any time to ensure survival under the sudden onset of adverse conditions.

The extracellular matrix (Figure 3) was consistently observed to be more abundant in all SCV samples compared with their corresponding control cells, a finding consistent with other studies [36,37]. Despite its prevalence, this feature was difficult to quantify. Nonetheless, the matrix may represent an augmented capacity for biofilm formation which could provide enhanced protection from a harsh environment [38,39].

The analyses of control and SCV cells *via* TEM revealed that the WT cells had thinner, well defined cell walls whereas, with the exception of the 4°C-treated *S. lugdunensis*, the SCV cell-walls were generally thicker and more diffuse (Figure 4). Thickened, more diffuse cell walls have been previously noted in staphylococci and in these instances it was suggested that the combination may serve as a protection against antibiotic penetration and enhance adherence for colonization purposes in device-related infections [40–42]. Following prolonged exposure to low-temperature stress, thicker, more diffuse cell-walls were correlated with altered amino acid composition in the cell-wall fractions extracted from *S. aureus*, *S.* epidermidis and *S. lugdunensis* [16]. Despite similar appearances on plate cultures, SCVs have different ultra-structural characteristics and implicit altered metabolic homeostasis to survive the specific variations of environmental stresses. The combination of a thicker and more diffuse cell-wall feature appears to be a more generic response to environmental stress factors.

The data presented in this study provided evidence that staphylococci can adapt to environmental stresses by altering their population characteristics with regard to cell size and cell-wall thickness. This can in turn lead to the formation of altered phenotypes to facilitate survival under harsh environmental conditions. The phenotypic response appeared to be governed by the type of environmental stress regime leading to appropriate alterations in ultra-structure and size which implies concomitant changes in gene expression, the proteome and metabolome. Understanding the mechanisms of control of these adaptive processes and the nature of the supporting metabolism that underlies the rapid responses for survival may provide avenues for generating new targets for generating antimicrobial chemicals.

## Supporting Information

File S1 Figure S1-S3: population distributions of *S. aureus*, *s.* epidermidis and *S. lugdunensis* WT and SCV comparisons following exposures to cold temperature, osmotic pressure, pH changes and antibiotics (Pen G and VA).
(DOCX)

Table S1 Data showing the mean cell sizes and numbers of sub-populations present in WT and SCV colony types of *S. aureus*, *s.* epidermidis and *S. lugdunensis*.
(DOCX)
