# Supplementary material for: Phenotypic Variants of Staphylococci and Their Underlying Population Distributions Following Exposure to Stress
Source: PLoS One. 2013 Oct 18;8(10):e77614. doi: 10.1371/journal.pone.0077614 (PMC3799968; doi:10.1371/journal.pone.0077614)
Supplement: Table S1 — Data showing the mean cell sizes and numbers of sub-populations present in WT and SCV colony types of S. aureus, s. epidermidis and S. lugdunensis. (DOCX) [file pone.0077614.s002.docx]

Table S1

| **Species** | **WT** | **PEN G** | **VA** | **TEMP** | **pH** | **NACL** |
| --- | --- | --- | --- | --- | --- | --- |
|  | **Mean cell sizes (nm) ± SE** | | | | | |
| *S. aureus* | 663.8±5.0 | 638.7±8.3 | 541.9±5.5 | 632.2±4.6 | 530.7±9.1 | 711.7±7.8 |
| *S. epidermidis* | 732.0±16.2 | 556.3±11.4 | 498.5±7.5 | 814.1±12.3 | 625.4±5.2 | 784.2±7.3 |
| *S. lugdunensis* | 616.3±5.9 | 582.9±6.8 | 531.1±3.8 | 543.6±10.8 | 532.4±4.7 | 486.1±11.2 |
|  |  |  |  |  |  |  |
